# *Specklinia dunstervillei*, a New Species Long Confused with *Specklinia endotrachys* (Orchidaceae: Pleurothallidinae)

**DOI:** 10.1371/journal.pone.0131971

**Published:** 2015-07-24

**Authors:** Adam P. Karremans, Franco Pupulin, Barbara Gravendeel

**Affiliations:** 1 Lankester Botanical Garden, University of Costa Rica, P.O. Box 302–7050, Cartago, Costa Rica; 2 Naturalis Biodiversity Center, Leiden University, Leiden, The Netherlands; 3 Harvard University Herbaria, Cambridge, Massachusetts, United States of America, and Marie Selby Botanical Gardens, Sarasota, Florida, United States of America; The National Orchid Conservation Center of China; The Orchid Conservation & Research Center of Shenzhen, CHINA

## Abstract

*Specklinia dunstervillei* is described as a new species in recognition of the distinctness of a Venezuelan species related to and confused with *Specklinia endotrachys*. It was illustrated for the first time by G. C. K. Dunsterville in 1963 from a plant collected in Trujillo on the Cordillera de Merida. The newly named species can be easily recognized by its small habit, short leaves and small reddish-orange flowers, the non-ascending dorsal sepal and the obtuse petals that are shortly apiculate. *Specklinia dunstervillei* is formally described and illustrated once again and compared morphologically and genetically with its closest relatives.

## Introduction

In the systematic study of the *Specklinia endotrachys* species complex, Pupulin et al. [1] found that, aside from the latter, *Specklinia pfavii* (Rchb.f.) Pupulin & Karremans, *S*. *remotiflora* Pupulin & Karremans and *S*. *spectabilis* (Ames) Pupulin & Karremans could all be recognized as distinct species in the complex. *Specklinia endotrachys* (Rchb.f.) Pridgeon & M.W.Chase had traditionally been considered a widely distributed and highly variable albeit morphologically quite unique species, nevertheless, the four proposed taxa could be easily distinguished morphologically and ecologically from each other. At the time, the authors recognized that not all the available material labeled *S*. *endotrachys* could be adequately placed into one of those four species concepts, and suspected more taxa would be recognized in the complex in the future [1,2,3].

The fifth species to be added to the complex was the Guatemalan *Specklinia juddii* (Archila) Pupulin & Karremans, unknown at the time of the first publication and later placed amongst its relatives [2,3]. It had been published under the generic name *Empusella* (Luer) Luer, a monotypic genus typified by *S*. *endotrachys* and here regarded as a synonym of *Specklinia* Lindl. [4,5,6]. Similarly, the Venezuelan material labeled *S*. *endotrachys* and illustrated for the first time in Dunsterville and Garay [7], morphologically did not resemble the other five species in the complex and had remained a mystery to the authors [1,2,3]. Now with additional material at hand we are able to compare the Venezuelan material with the other species of this complex, both morphologically (for all species currently recognized) and genetically (for all except *S*. *juddii*).

## Materials and Methods

This study was conducted at Jardín Botánico Lankester (JBL) of the Universidad de Costa Rica and Naturalis Biodiversity Center—Leiden University, from 2012 to 2014. Plants were collected under the scientific permits handed by the Costa Rican Ministry of Environment (MINAE) to researchers at JBL. Individual plants were photographed, illustrated and preserved as DNA samples, herbarium specimens and spirit specimens in formaldehyde: acetic acid: ethanol [FAA (53% ethanol, 37% water, 5% formaldehyde and 5% glycerol)] (only including flowers) for future reference, deposited at JBL-spirit and L-spirit and the DNA bank of Naturalis Biodiversity Center. Taxon names mostly follow Pridgeon [8].

### Photography

The Lankester Composite Digital Plate (LCDP) and color illustrations of the flowers were made using a Nikon D5100 digital camera with a AF-S VR Micro-NIKKOR 105mm f/2.8G IF-ED lens and an Epson Perfection Photo Scanner V600, and a Leica MZ9.5 stereomicroscope fitted with a Leica DFC295 digital microscope color camera with Leica FireCam 3.4.1 software.

### Phylogenetic analysis

The data matrix included DNA sequences of 50 individuals (Table 1), 27 of which were produced in this study. The remaining data were obtained from GenBank [4,5,9]. Fresh leaf and flower cuttings of approximately 1 cm^2^ were dried with silica gel. Samples (20 mg) were pulverized and extraction performed following the DNEasy procedure (Qiagen). The nuclear ribosomal internal transcribed spacer (nrITS) region was amplified using the methods and primers for sequencing and amplification described by Sun et al. [10], and Sanger sequencing was done commercially by Macrogen on a 96-capillary 3730xl DNA Analyzer automated sequencer (Applied Biosystems, Inc.) using standard dye-terminator chemistry (Macrogen, Inc.).

**Table 1:** List of the 50 accessions used in the phylogenetic analysis. The vouchers, NCBI GenBank accession number and source are given. Scientific names follow Pridgeon 2005.

Taxon	Sequence Voucher	GenBank Accession Number	Sequence Source
*Dryadella simula* (Rchb. f.) Luer	*Chase 1095*	AF262825	Pridgeon et al. [4]
*Dryadella susanae* (Pabst) Luer	*Chiron 11240*	JQ306486	Chiron et al. [9]
*Phloeophila peperomioides* (*Ames*) Garay	*None*	AF275690	Pridgeon et al. [4]
*Platystele compacta* (Ames) Ames	*Chase 5637*	AF262822	Pridgeon et al. [4]
*Platystele misera* (Lindl.) Garay	*Chase 5625*	AF262823	Pridgeon et al. [4]
*Platystele stenostachya* (Rchb. f.) Garay	*Chase 5618*	AF262821	Pridgeon et al. [4]
*Scaphosepalum grande* Kraenzl.	*Chase 1107*	AF262819	Pridgeon et al. [4]
*Scaphosepalum swertiifolium* (Rchb. f.) Rolfe	*Chase 1383*	AF262818	Pridgeon et al. [4]
*Scaphosepalum verrucosum* (Rchb. f.) Pfitzer	*Chase 1331*	AF262820	Pridgeon et al. [4]
*Specklinia absurda* Bogarín, Karremans & Rincón	*Bogarín 8711 (JBL-Spirit)*	KC425827	Bogarín et al. [5]
*Specklinia barbae* (Schltr.) Luer	*Karremans 4853*	KC425771	This Study
*Specklinia barbae* (Schltr.) Luer	*Karremans 3928*	KC425769	This Study
*Specklinia chontalensis* (A.H.Heller & A.D.Hawkes) Luer (1)	*Pupulin 6543*	KC425776	This Study
*Specklinia chontalensis* (A.H.Heller & A.D.Hawkes) Luer (2)	*Pupulin 6543*	KF747799	This Study
*Specklinia costaricensis* (Rolfe) Pridgeon & M.W.Chase	*Chase 5612*	AF262862	Pridgeon et al. [4]
*Specklinia digitalis (Luer) Pridgeon & M*.*W*.*Chase*	*Karremans 5737*	KF747806	This Study
*Specklinia dunstervillei*	*Karremans 5966*	KP012456	This Study
*Specklinia endotrachys* (Rchb.f.) Pridgeon & M.W.Chase (1)	*Blanco 961*	KC425784	This Study
*Specklinia endotrachys* (Rchb.f.) Pridgeon & M.W.Chase (2)	*Blanco 961*	KF747810	This Study
*Specklinia fuegi* (Rchb.f.) Solano & Soto Arenas	*Karremans 5600 (JBL-Spirit)*	KC425786	Bogarín et al. [5]
*Specklinia fulgens* (Rchb.f.) Pridgeon & M.W.Chase	*Chase 5630*	AF262872	Pridgeon et al. [4]
*Specklinia glandulosa* (Ames) Pridgeon & M.W.Chase	*Karremans 5501*	KC425792	This Study
*Specklinia glandulosa* (Ames) Pridgeon & M.W.Chase	*Karremans 3265*	KC425791	This Study
*Specklinia glandulosa* (Ames) Pridgeon & M.W.Chase	*Karremans 2945*	KP012452	This Study
*Specklinia glandulosa* (Ames) Pridgeon & M.W.Chase	*Karremans 5944*	KP012453	This Study
*Specklinia glandulosa* (Ames) Pridgeon & M.W.Chase	*Bogarín 2895*	KP012454	This Study
*Specklinia glandulosa* (Ames) Pridgeon & M.W.Chase	*Karremans 3268*	KP012455	This Study
*Specklinia grobyi* (Bateman ex Lindl.) F.Barros	*Chiron 04524*	JQ306485	Chiron et al. [9]
*Specklinia lanceola* (Sw.) Lindl. (2)	*Pridgeon s*.*n*.	KC425838	Bogarín et al. [5]
*Specklinia lanceola* (Sw.) Lindl. (3)	*Chase 1433*	AF262861	Pridgeon et al. [4]
*Specklinia lentiginosa* (F.Lehm. & Kraenzl.) Pridgeon & M.W.Chase		AF275692	Pridgeon et al. [4]
*Specklinia montezumae* (Luer) Luer	*Karremans 229 (JBL-Spirit)*	KC425811	Bogarín et al. [5]
*Specklinia montezumae* (Luer) Luer	*Karremans 5751*	KF747816	This Study
*Specklinia picta* (Lindl.) Pridgeon & M.W.Chase	*Van Den Berg 2146*	JQ306384	Chiron et al. [9]
*Specklinia pissina*	*Karremans 4797*	KC425795	This Study
*Specklinia pissina*	*Karremans 4839*	KC425797	This Study
*Specklinia pfavii* (Rchb.f.) Pupulin & Karremans	*Karremans 4825*	KC425814	This Study
*Specklinia pfavii* (Rchb.f.) Pupulin & Karremans	*Karremans 3656*	KF747819	This Study
*Specklinia pfavii* (Rchb.f.) Pupulin & Karremans	*JBL-11086*	KF747820	This Study
*Specklinia remotiflora* Pupulin & Karremans (4)	*Chase 1303*	AF262859	Pridgeon et al. [4]
*Specklinia remotiflora* Pupulin & Karremans (1)	*Karremans 4798a*	KC425818	This Study
*Specklinia remotiflora* Pupulin & Karremans (2)	*Karremans 4798b*	KC425819	This Study
*Specklinia remotiflora* Pupulin & Karremans (3)	*Karremans 4854*	KC425820	This Study
*Specklinia sp*.	*Karremans 6025*	KP012457	This Study
*Specklinia sp*.	*Pupulin 7709*	KC425824	This Study
*Specklinia spectabilis* (Ames & C.Schweinf.) Pupulin & Karremans	*Bogarín 7401*	KC425830	This Study
*Specklinia spectabilis* (Ames & C.Schweinf.) Pupulin & Karremans	*Karremans 5699*	KC425828	This Study
*Specklinia subpicta* (Schltr.) F.Barros	*Chiron 11046*	JQ306389	Chiron et al. [9]
*Specklinia succulenta* Bellone & Archila	*Bellone 680*	JQ306383	Chiron et al. [9]
*Specklinia tribuloides* (Sw.) Pridgeon & M.W.Chase (1)	*Chase 5615*	AF262867	Pridgeon et al. [4]

The Staden et al. [11] package was used for editing of the sequences. Contigs were exported as.fas files and opened in Mesquite v2.72 (Maddison & Maddison [12]), where they were checked for base calling errors, the matrix was aligned manually (S1 File: Sequence Matrix). The ends of each data set were trimmed to eliminate possible erroneous data, and gaps were regarded as missing data (filled with Ns). *Phloeophila peperomioides* was used as the outgroup, as it was found to be one of the most distantly related of all included species in this phylogenetic analysis (Pridgeon *et al*. [4]). The trees were produced with an analysis of the nrITS dataset of 43 sequences using BEAST v1.6.0. (Drummond & Rambaut [13]). Parameters were set to preset, except for substitution model GTR with 10 categories, clock model uncorrelated lognormal, tree prior Yule process, and number of generations 20,000,000. The resulting trees were combined using TreeAnnotator v1.6.0., where the first 3000 trees were used as burn-in. FigTree v1.3.1. (Rambaut [14]) was used to edit the resulting tree. Posterior probabilities are given for each node in decimal form.

A pairwise comparison of the ITS sequence of the accessions of *S*. *dunstervillei*, *S*. *endotrachys*, *S*. *montezumae* (as an outgroup), *S*. *pfavii*, *S*. *spectabilis* and *S*. *remotiflora* is presented in Table 2. Each different base was counted as an individual change, even when concurrent; insertions and deletions were counted as a single change regardless of length. All the mentioned accession of each species in Table 1 were combined and used for the comparison except for AF262859, a sequence labeled *S*. *endotrachys* by Pridgeon et al. [4] but which we suspect (based on DNA data) should be *S*. *remotiflora* or a closely related species.

**Table 2:** Pairwise comparison of the number of single base differences amongst the nrITS sequences of *S*. *dunstervillei*, *S*. *endotrachys*, *S*. *montezumae*, *S*. *pfavii*, *S*. *spectabilis* and *S*. *remotiflora*.

	*S*. *dunstervillei*	*S*. *endotrachys*	*S*. *pfavii*	*S*. *remotiflora*	*S*. *spectabilis*	*S*. *montezumae*
*Specklinia dunstervillei*	-	2	4	10	2	10
*Specklinia endotrachys*	2	-	4	10	0	10
*Specklinia pfavii*	4	4	-	12	4	11
*Specklinia remotiflora*	10	10	12	-	10	9
*Specklinia spectabilis*	2	0	4	10	-	10
*Specklinia montezumae*	10	10	11	9	10	-

### Nomenclature

The electronic version of this article in Portable Document Format (PDF) in a work with an ISSN or ISBN will represent a published work according to the International Code of Nomenclature for algae, fungi, and plants, and hence the new names contained in the electronic publication of a PLOS ONE article are effectively published under that Code from the electronic edition alone, so there is no longer any need to provide printed copies.

## Results

### Photography

**Fig 1:**
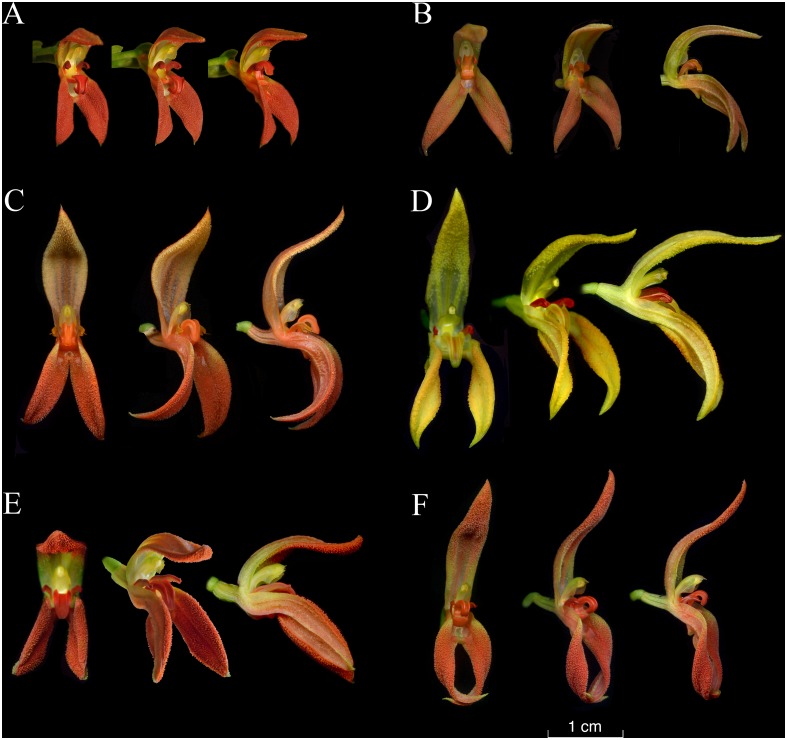
Comparison of flowers of species of the *Specklinia endotrachys* group. A. *Specklinia dunstervillei* (*Karremans 5966*). B. *Specklinia dunstervillei* (*Karremans 5899*). C. *Specklinia endotrachys* (*Blanco 961*). D. *Specklinia pfavii* (*JBL-11086*). E. *Specklinia remotiflora* (*Bogarín 8181*). E. *Specklinia spectabilis* (*JBL-02641*). All flowers shown in front, three-quarters side, and side views. Photographs by F. Pupulin (B-F) and R. van Vugt (A).

The color illustrations of species of the *Specklinia endotrachys* complex (Fig 1) shows a morphologically distinct entity, *Specklinia dunstervillei* (Fig 1A and 1B), recognized amongst others by the smaller flowers and shortly apiculate petals.

### Phylogenetic analysis

**Fig 2:**
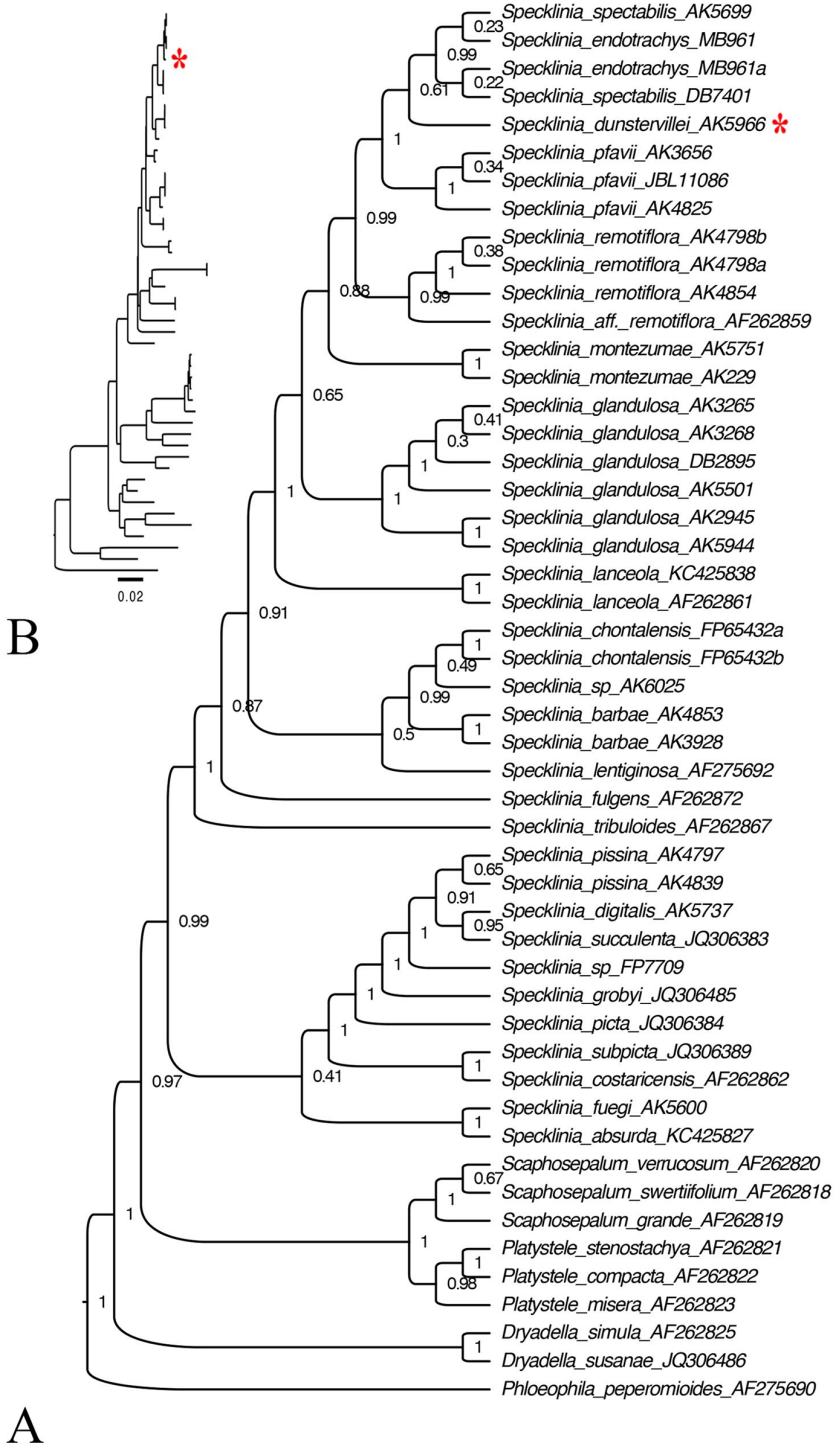
Phylogenetic relationship amongst the species of the *Specklinia endotrachys* complex. The trees were produced with an analysis of the nrITS dataset of 50 sequences using BEAST v1.6.0. Node values are posterior probabilities. The tree was edited using FigTree v.1.3.1. A. Tree with branches transformed to be of equal length. B. Branch lengths relative to relative number of changes.

The consensus gene tree (Fig 2) was obtained from a BEAST analysis of a matrix of 45 ITS sequences (Table 1), including 12 accessions belonging to 5 different species of the *Specklinia endotrachys* complex. The accessions of *S*. *dunstervillei*, *S*. *endotrachys*, *S*. *pfavii*, *S*. *spectabilis* and *S*. *remotiflora* are found in a highly supported monophyletic clade (P.P. = 0.99), sister to the accessions of *S*. *montezumae*.

The pairwise comparison of the ITS sequence of the accessions of the *S*. *endotrachys* species complex (Table 2) shows that the sequences of *Specklinia endotrachys* and *S*. *spectabilis* do not differ from each other, while *S*. *dunstervillei* differs in 2 bases from those species. *Specklinia pfavii* and *S*. *remotiflora* differ in 4 and 10 bases respectively, from the three before mentioned species. *Specklinia montezumae* differs in 9 to 11 bases from each of the members of the *S*. *endotrachys* species complex.

## Discussion

Considering all the available evidence, including morphology, genetics, distribution and ecology, we find that the Venezuelan material labeled *Specklinia endotrachys*, actually belongs to an unnamed species, described here forth:

### *Specklinia dunstervillei* Karremans, Pupulin & Gravendeel, sp. nov.

[urn:lsid:ipni.org:names: 77147597–1]

#### Type

**Fig 3:**
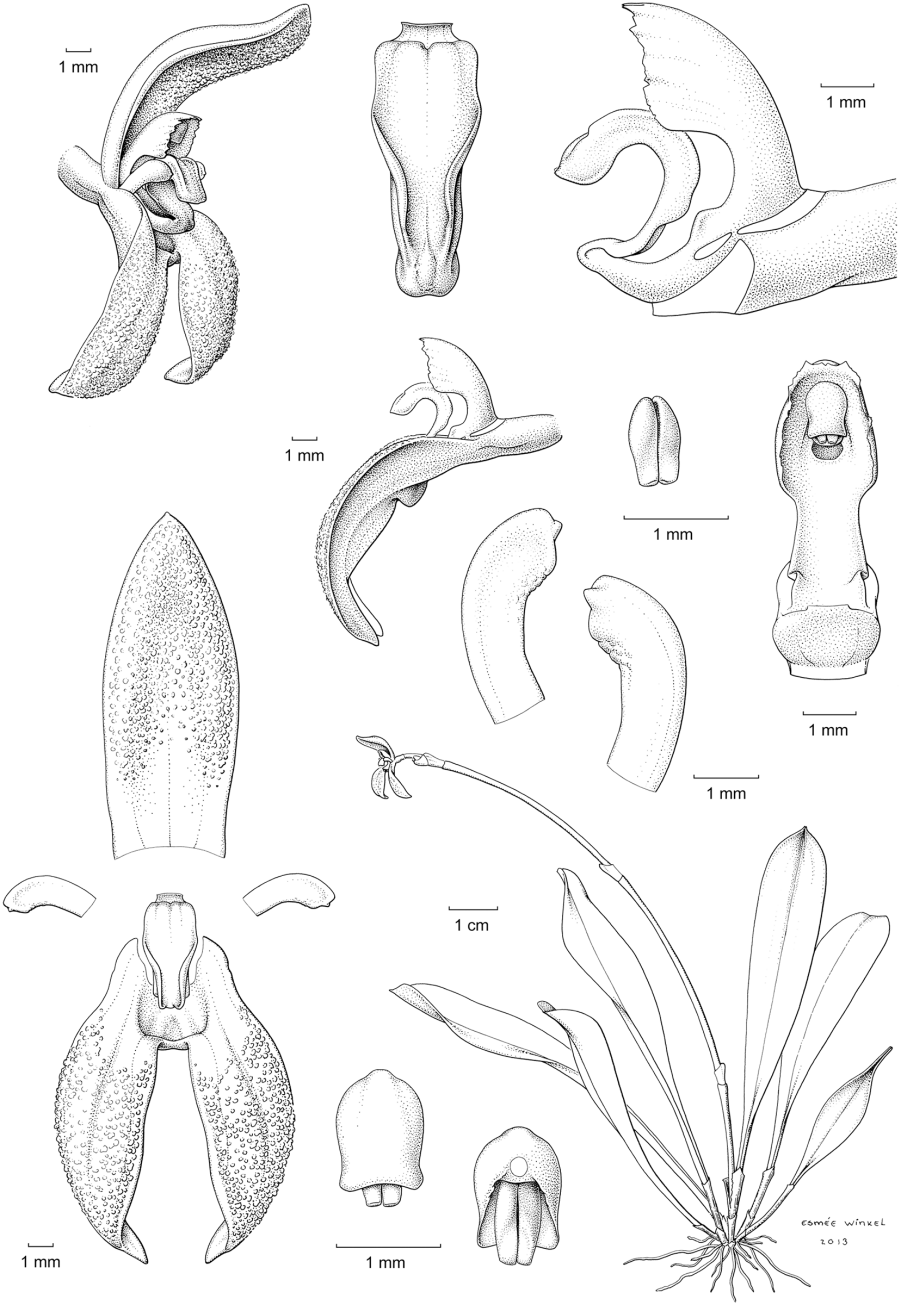
*Specklinia dunstervillei* Karremans, Pupulin & Gravendeel . A) Habit; B) Flower; C) Dissected perianth; D) Lateral view of the lip placement relative to lateral sepals; E) Column and lip, lateral view; F) Column, ventral view; G) Lip, extended; H) Petals; I) Anther cap with pollinia; J) Pollinia. Drawn from the holotype (*Karremans 5966*) by Esmée Winkel.

Venezuela. Without collecting data, cultivated by Jacobus Wubben in the Netherlands. Flowered in cultivation on March 29^th^ 2013, *A*.*P*. *Karremans 5966 & B*. *Gravendeel* (holotype, JBL-spirit!; isotype, L-spirit!; Figs 1 and 3).

The species is similar to Specklinia endotrachys but can be distinguished by the small habit, shorter leaves, smaller flowers, the flat, non-ascending dorsal sepal (vs. ascending), and the obtuse, shortly apiculate (vs. emarginate and long mucronate) petals.

Epiphytic, caespitose, erect herb to 10 cm tall, excluding the inflorescence. Roots fibrous, flexuous, glabrous. Stem abbreviated, terete-cylindric, to 1 cm long, monophyllous, completely concealed by papyraceous, subancipitous, acute sheaths to 1 cm long. Leaf narrowly obovate-oblanceolate, 6–10 x 1.2–1.8 cm, minutely emarginate at apex, the mid-vein protruding abaxially into a small apicule, gradually tapering toward the base into a deeply conduplicate petiole with strongly ancipitous margins, subcoriaceous. Inflorescence borne laterally from the apex of the stem, without an annulus, a distichous, successively flowered raceme, with only one flower open at once, up to 12–13 cm long; peduncle flattened, ancipitous, to 11–12 cm long, with 2–3 amplectent, ancipitous, subacute bracts, 5–6 mm long. Floral bracts infundibuliform, broadly ovate, acute to subacuminate, 2–3 mm long. Pedicel cylindric, glabrous, 4 mm long, persistent. Ovary subclavate, 2 mm long, green tinted with orange. Flowers with reddish-orange sepals, petals and lip, the column greenish-yellow, lightly tinted orange. Sepals fleshy, densely papillose in the inner surface except at the base; dorsal sepal elliptic, 3-veined, acute, the base whitish semi-hyaline, flushed with orange along the veins, the distal two thirds densely papillose, 14–16 × 5–6 mm; lateral sepals narrowly elliptic-oblanceolate, subfalcate, 3–veined, 13–15 × 4 mm, the base saccate, membranaceous-hyaline, the apex acute, gently twisting above the middle, the midvein strongly carinate abaxially. Petals small, ligulate-falcate, truncate, shortly apiculate, porrect, 3–4 × 1 mm, 1-veined, papillose-thickened toward the concave apex. Lip small, longitudinally arched-convex in natural position, thinly articulate with the column foot by a hyaline claw, narrowly elliptic-lanceolate when expanded, obtuse, the apex reflexed, appearing minutely retuse, the clawed base thickened, transversely minutely gibberose, 5–6 × 2 mm, provided with 2 slender keels, fringed-lacerate at the base, gently converging from the base of the lamina to near the apex. Column arched, terete-slender at the base, 4.5–5.0 mm long without the foot, provided with broad membranous wings serrulate along the margins, at the apex forming a deeply cucullate, serrulate clinandrium; column foot forward-projecting, stout, fleshy, incurved, 1.5 mm long. Anther cap deeply cucullate, ovate, 2-celled. Pollinia 2, obovate-complanate, minutely hooked at the base, lacking caudicles.

*Note*: Only the specimens from Venezuela were used for the description (*Dunsterville 757* and *Karremans 5966*).

#### Etymology

The name honors G. C. K. Dunsterville, who prepared the first known illustration of the species.

#### Additional material examined

**Fig 4:**
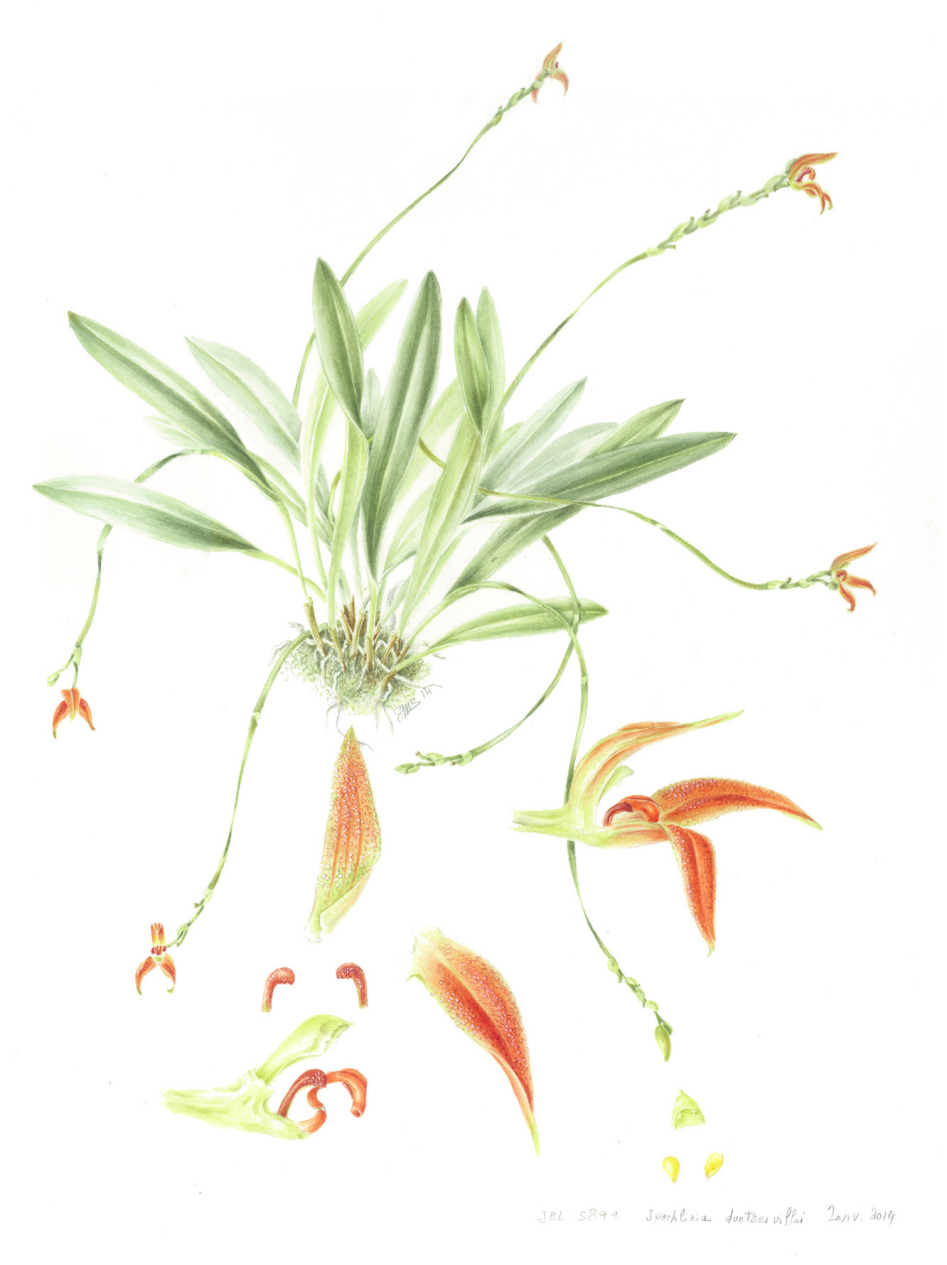
Acuarela of *Specklinia dunstervillei* Karremans, Pupulin & Gravendeel. By Sylvia Strigari, based on *Karremans 5899* (JBL).

**Fig 5:**
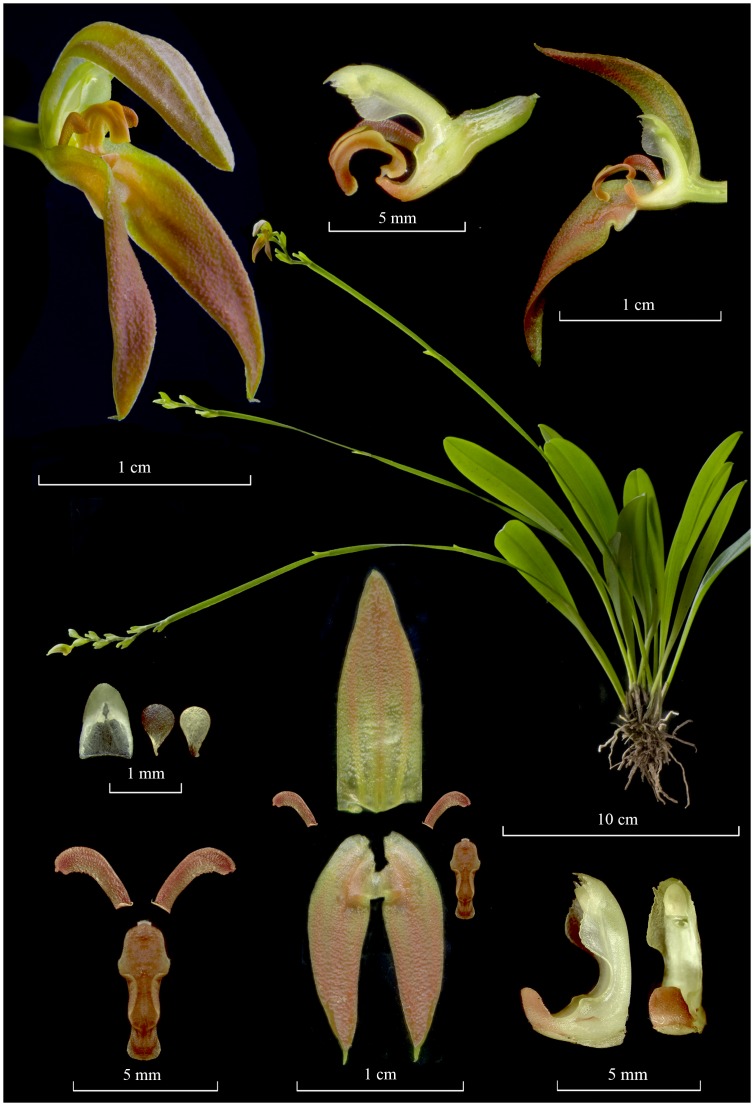
Lankester Composite Dissection Plate (LCDP) of *Specklinia dunstervillei* Karremans, Pupulin & Gravendeel. A. Habit. B. Flower. C. Transverse section of a whole flower. D. Dissected perianth. E. Column and lip, lateral view. F. Petals and lip. G. Column in ventral and lateral view. H. Pollinia and anther cap. Based on photographs of *Karremans 5899* (JBL) by A.P. Karremans.

**Fig 6:**
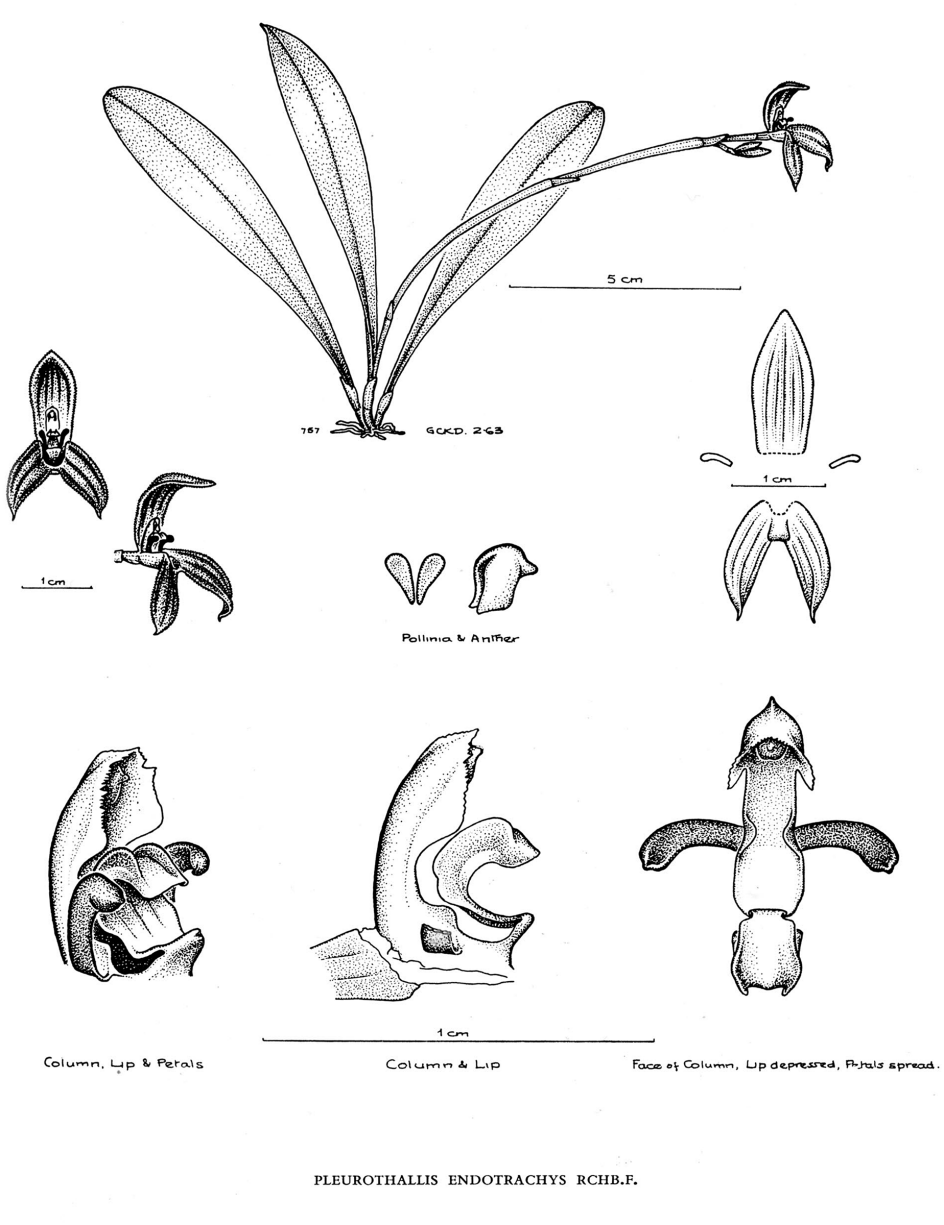
*Specklinia dunstervillei* Karremans, Pupulin & Gravendeel. Illustrated by G. C. K. Dunsterville in Dunsterville and Garay [7] from a plant found in Trujillo, Venezuela (*Dunsterville 757*). Reproduced with the kind permission of the Orchid Herbarium of Oakes Ames, the Harvard University Herbaria.

**Fig 7:**
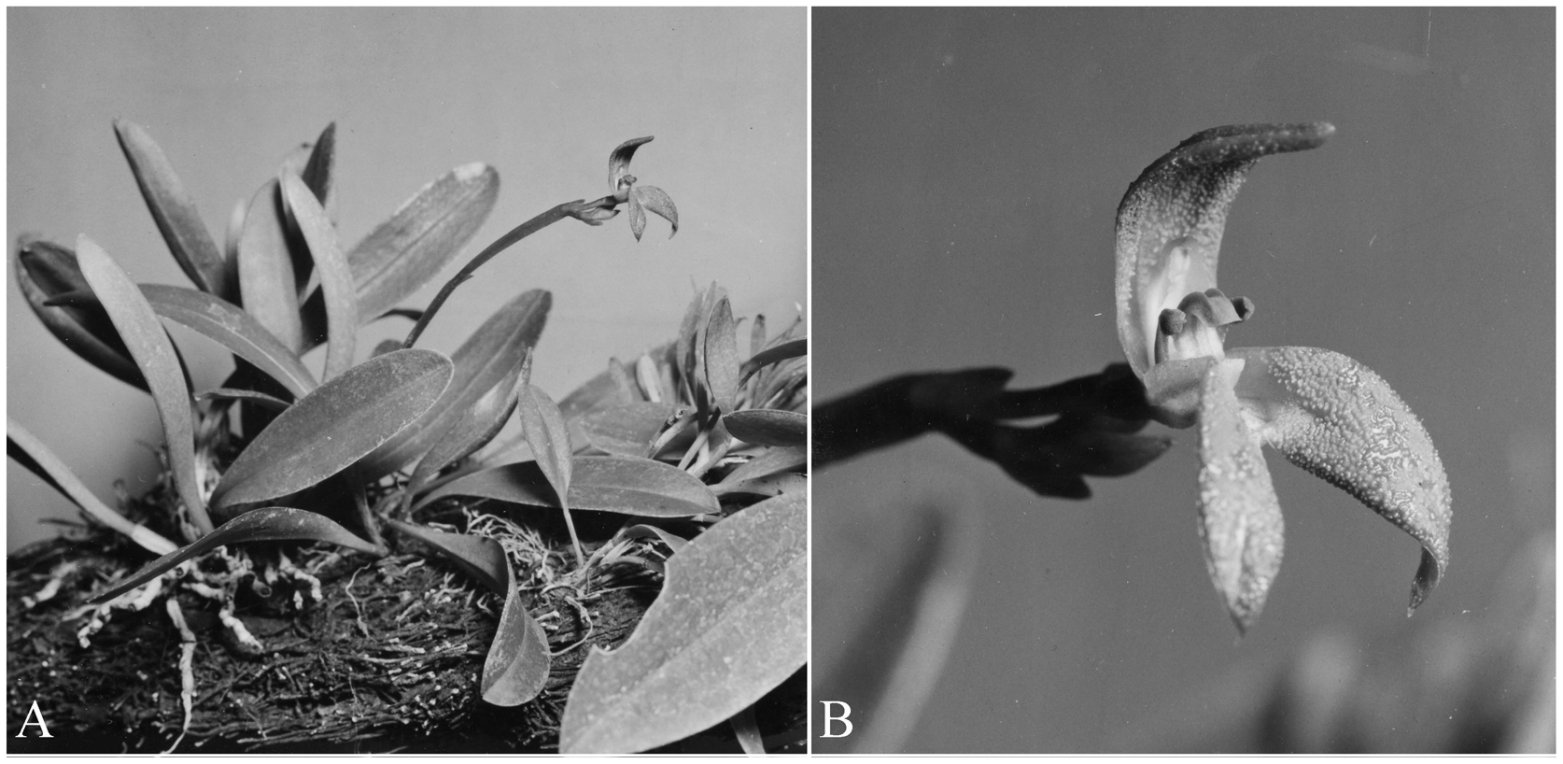
*Specklinia dunstervillei* Karremans, Pupulin & Gravendeel. Unpublished photographs by G. C. K. Dunsterville from the plant found in Trujillo, Venezuela (*Dunsterville 757*). A. The plant habit. B. Detail of the flower. Reproduced with the kind permission of the Orchid Herbarium of Oakes Ames, the Harvard University Herbaria.

Costa Rica. Without collecting data, cultivated by Gerson Villalobos in Moravia, San José. Flowered in cultivation on September 1^st^ 2013, *A*.*P*. *Karremans 5899* (JBL-spirit!; Figs 4 and 5). Venezuela. Boconó-Guaramacal penetration road. About 8000 ft. in rain forest, *G*. *C*. *K*. *Dunsterville 757* (illustration of voucher in Dunsterville and Garay [7]!; Figs 6 and 7).

#### Other records

Colombia. Without collecting data, illustration of voucher in Ortiz and Uribe [15].

#### Distribution

The material collected by Dunsterville in Venezuela comes from the road between Boconó and Guaramacal in Trujillo, on the Cordillera de Merida, a branch of the Colombian Andes, at an elevation of about 2400 m. A few specimens that have been found in private collections in Costa Rica and Colombia lack precise location data.

#### Key to the species of the empusellous species of *Specklinia*

1. Flowers yellow with red petals; lip acute, yellow, with a central red line; column wings broad, entire; clinandrium entire ........................................................................................................ *S*. *pfavii*
1. Flowers reddish-orange; lip rounded to truncate, concolorous orange; column wings narrow or broad, erose-denticulate; clinandrium erose-dentate .......................................................................... 2
2. Flowers campanulate, with lateral sepals straight, not spreading, petals obtuse ............................ 3
3. Plant repent, inflorescence lax; flowers bright orange in both outer and inner surfaces; column with rectangular wings and erose-dentate clinandrium .................................................. *S*. *remotiflora*
3. Plant caespitose, inflorescence congested; flowers green outside, orange brown within; column with broadly elliptic wings and entire clinandrium ................................................................. *S*. *juddii*
2. Flowers spreading, lateral sepals twisted and/or bent, petals apiculate to mucronate ................... 4
4. Plant and flowers small (leaf up to 10 cm long, dorsal sepal about 1.5 cm long), dorsal sepal bent forward, but not twisted, petals shortly apiculate ........................................ *S*. *dunstervillei*
4. Plant and flowers larger (leaf longer than 10 cm long and sepals at least 2 cm long), dorsal sepal erect, ascending, petals long-mucronate ......................................................................... 5
5. Floral bracts subequal to the length of the pedicel; dorsal sepal lanceolate, lateral sepals bent but not twisted; petals retuse, with a long mucron abruptly inserted within the sinus ....... *S*. *endotrachys*
5. Floral bracts much shorter than the length of the pedicel; dorsal sepal linear-triangular, lateral sepals twisted; petals acute, tapering, the mucron continuous with the apex ................. *S*. *spectabilis*

#### Ecology

The typical form of *Specklinia dunstervillei* is that found in Colombia and Venezuela (Figs 1a, 3 and 6). The material illustrated by Dunsterville, which lacks an herbarium voucher (Gustavo Romero pers. comm.), was collected on the Cordillera de Merida at about 2400 m in elevation. That makes the new species the one with the most Easternly distribution in the complex and, together with *S*. *remotiflora*, the only one to grow in cloud forests at high elevations above 1800 m. A photograph of this species was published in Ortiz and Uribe [15] without precise locality, nonetheless it was definitively taken from a Colombian plant (Carlos Uribe V. pers. comm.). Considering that the Cordillera de Merida extends into Colombia, it is indeed not surprising that the species is also found there. Finally, we have chosen to regard the material found in Costa Rican private collections as *S*. *dunstervillei* as they are morphologically most similar to that species concept, nevertheless they do show quite some variation (Figs 1b, 4 and 5). The studied Costa Rican specimens lack specific collection data.

**Fig 8:**
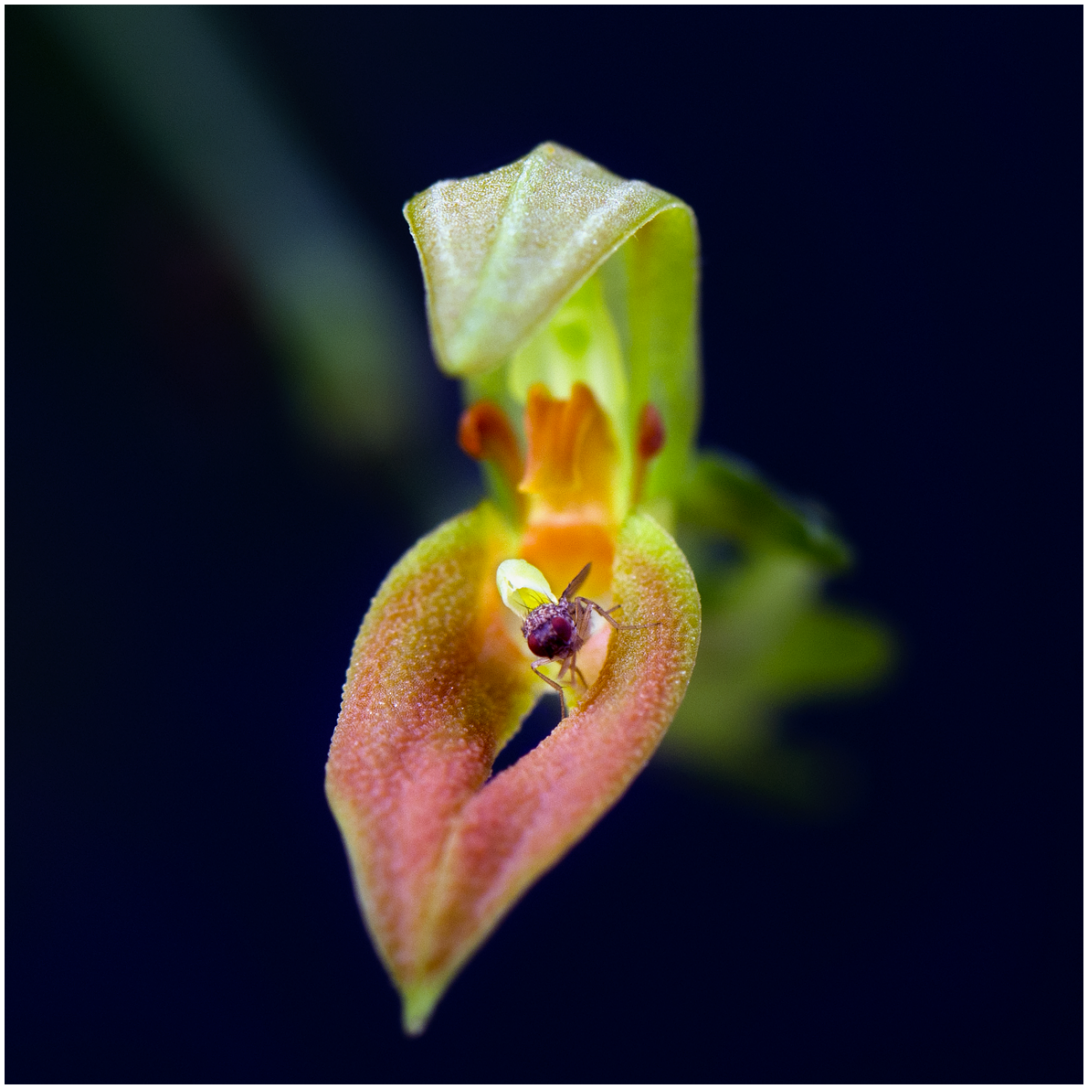
*Drosophila* sp. adpressed by the lip against the column of *Specklinia dunstervillei* Karremans, Pupulin & Gravendeel *(Karremans 5899)*. Photographed by A.P. Karremans.

Like the other species of the *S*. *endotrachys* complex, *S*. *dunstervillei* has nectar secreting stomata placed on the apex of the warts that cover the surface of the sepals [1,2,3,6]. The released nectar gives the sepals a wet-glossy appearance, as noted by Dunsterville and Garay [7], and is given special attention by visiting fruit flies. At Lankester Botanical Garden the flowers were frequently visited by up to 6 individuals of *Drosophila* spp. The flies wander about on the sepals, sucking on the papillae rich surfaces, occasionally removing the pollinia, after stepping on the movable lip and being adpressed against the column (Fig 8).

#### Morphological recognition

*Specklinia dunstervillei* is morphologically similar to other members of the *S*. *endotrachys* complex. Within *Specklinia* they are recognized by the relatively large habit and flowers, the long, successive, flat, ancipitous inflorescence, the bright yellow to orange-red flowers, the verrucose sepals, the relatively minuscule petals and the highly sensitive strongly concave lip. Amongst its closest relatives, *S*. *dunstervillei* is recognized by the tiny habit, caespitose plants, short leaves (up to about 10 cm), the small flowers (dorsal sepal 14–16 × 5–6 mm), the flattening, non-ascending dorsal sepal, the lateral sepals that spread, slightly twisting downwards and the obtuse petals, which are shortly apiculate.

#### Genetic recognition

The nrITS sequence of the accession of *Specklinia dunstervillei* differs in two base pairs (out of just under 800bp) from the accessions of both *S*. *endotrachys* and *S*. *spectabilis*, which are identical to each other (Table 2). Considering that the species of the *S*. *endotrachys* complex differ in only 9 to 11 bases (between 1.1 and 1.4%) from an outgroup as morphologically distinct as *S*. *montezumae*, it becomes highly plausible that species with significant morphological and ecological differences within the complex itself, such as *S*. *endotrachys* and *S*. *pfavii*, can differ in just about 0.5% bases. Therefore it is also not unusual that more closely related species such as *S*. *endotrachys* and *S*. *dunstervillei* differ in only 2 bases, or that the sisters *S*. *endotrachys* and *S*. *spectabilis* do not differ at all in this particular DNA barcode.

The internal transcribed spacer (ITS) of nuclear ribosomal DNA has consistently shown high levels of discrimination among many species of plants [16] including Pleurothallidinae species, and are well suited for a broad range of phylogenetic studies [4,10,17,18]. However, in current literature DNA data are mostly used to support so called “cryptic” novel taxa, when the accession of a said taxon does not cluster with other accessions of the *a priori* believed same taxon [19,20,21,22]. When different accessions of a species complex cluster together authors seem to believe that there is evidence that they are a single species, however that is not only an illogical conclusion but also frequently untrue. Be it accessions of the same species, or sister species, or species of a single genus or the same family, any two accessions will cluster together with high support if they are relatively much more similar to each other than to the other sequences analyzed. The only measurable difference between the mentioned sister pairs would be the branch length (as a measure of number of base pair changes). However, as there is no established similarity threshold above which one should consider two sequences to belong to two different species, or below which they should be considered a single species, it is at this time also not possible to infer from branch lengths alone if we are dealing with a single or more than one species. Meyer and Paulay [23] found that this especially counts for taxonomically understudied groups that are not yet thoroughly sampled.

In Pleurothallidinae, DNA data have been seldomly used to support novel species descriptions. Ramos-Castro et al. [24] used an nrITS based phylogeny to prove that their novel taxon *Stelis zootrophionoides* Castañeda-Zárate & Ramos-Castro belonged in that genus, however they did not venture into using DNA data to set their novelty apart from other taxa in the genus. In the phylogeny presented, the single accession of *S*. *zootrophionoides* clusters, as would be expected, with that of the morphologically similar *S*. *nigriflora* (L.O.Williams) Pridgeon & M.W.Chase. Therefore, their phylogeny by itself could not prove that the accessions belonged to two different species, and the authors relied exclusively on morphological differences to establish their novelty. A species labeled *Specklinia* sp. in Chiron et al. [9], of which the nrITS sequence clustered with that of *Specklinia marginata* (Lindl.) Pridgeon & M.W.Chase, was later published as a distinct species using mainly morphological characters. In both cases the authors could also have argued that the nrITS sequence of the accession of their novelty differed in about 1% from the sequence of the accession of its closest relative with which it clustered. A different approach was that of Meyer et al. [25], who used the presence of insertions/deletions in the *trnH*-*psbA* and *ycf1* sequences of accessions of *Dracula radiosa* (Rchb.f.) Luer and close relatives to support the establishment of their novel species *Dracula trigonopetala* Gary Mey. & L. Baquero. In that case their novelty differed in less than 0.3% from its closest relatives (as an insertion/deletion counts for a single change), however, the 26 bp unique insertion in the *trnH*-*psbA* sequence of their novelty stands out in an otherwise quite conserved region and therefore easily sets it apart from its close relatives.

## Conclusions

A common misconception amongst modern authors is that DNA data will in itself resolve taxonomic issues. In fact, that two sequences are identical in a particular region does not guarantee that they belong to the same species, nor does the fact that they differ guarantee that they belong to different species. DNA data albeit less subjective than morphological characterization, are nonetheless subject to many of the same pitfalls, which can basically be summarized as: 1) interpretation of the type specimens and correct application of names; 2) reduction or elimination of data reading mistakes; 3) adequate interpretation of the observed variation.

Considering all the evidence at hand we conclude that the specimen illustrated in Dunsterville and Garay [7] from Venezuela and labeled *Specklinia endotrachys* actually represents an until now unnamed species. With the inclusion of the here proposed novelty, *Specklinia dunstervillei*, the *S*. *endotrachys* species complex now consists of six distinctly named species, instead of a single variable species. Its highest diversity is found in Costa Rica and Panama, but this might change as additional material becomes available from less sampled areas; we therefore do not reject the possibility of discovering other novelties in this group in the future.

## Supporting Information

S1 File Alignment matrix of 43 nrITS sequences used to infer the molecular phylogeny presented in Fig 2. The ends were trimmed and gaps were regarded as missing data.
(NEX)

## References

[1] Pupulin F, Karremans AP, Gravendeel B (2012) A reconsideration of the empusellous species of *Specklinia* (Orchidaceae: Pleurothallidinae) in Costa Rica. Phytotaxa 63: 1–20.

[2] Pupulin F, Karremans AP, Strigari S (2013) Taxonomie in Aquarell: die *Specklinia*-*endotrachys*-Gruppe, Teil 1. Taxonomy in watercolor: the *Specklinia endotrachys* group, part 1. Die Orchidee (Hamburg) 64(5): 392–399.

[3] Pupulin F, Karremans AP, Strigari S (2013) Taxonomie in Aquarell: die *Specklinia*-*endotrachys*-Gruppe, Teil 2. Taxonomy in watercolor: the *Specklinia endotrachys* group, part 2. Die Orchidee (Hamburg) 64(6): 475–485.

[4] Pridgeon AM, Solano-Gómez R, Chase MW (2001) Phylogenetic relationships in Pleurothallidinae (Orchidaceae): combined evidence from nuclear and plastid DNA sequences. Am. J Bot 88(12): 2286–2308. 21669661

[5] Bogarín D, Karremans AP, Rincón R, Gravendeel B (2013) A new *Specklinia* (Orchidaceae: Pleurothallidinae) from Costa Rica and Panama. Phytotaxa 115(2): 31–41.

[6] Karremans AP, Pupulin F, Gravendeel B (2013) Taxonomy, molecular phylogenetics, reproductive isolation, and niche differentiation of the *Specklinia endotrachys* species complex (Orchidaceae: Pleurothallidinae). Lankesteriana 13(1–2): 132–133.

[7] Dunsterville GCK, Garay LA (1965): Venezuelan Orchids Illustrated 3. London: Andre Deutsch Ltd.

[8] Pridgeon AM (2005) Subtribe Pleurothallidinae In: Prigeon AM, Cribb PJ, Chase MW, Rasmussen FN, editors, Genera Orchidacearum. Volume 4 Epidendroideae (Part One). Oxford: Oxford University Press pp. 319–422.

[9] Chiron GR, Guiard J, van den Berg C (2012) Phylogenetic relationships in Brazilian *Pleurothallis* sensu lato (Pleurothallidinae, Orchidaceae): evidence from nuclear ITS rDNA sequences. Phytotaxa 46: 34–58.

[10] Sun Y, Skinner DZ, Liang GH, Hulbert H (1994) Phylogenetic analysis of *Sorghum* and related taxa using internal transcribed spacers of nuclear ribosomal DNA. Theor. Appl. Genet. 89: 26–32. 10.1007/BF00226978 24177765

[11] Staden R, Judge DP, Bonfield JK (2003) Analysing Sequences Using the Staden Package and EMBOSS In: Krawetz S. A. and Womble D. D. (Eds.), Introduction to Bioinformatics. A Theoretical and Practical Approach. Human Press Inc., Totawa, NJ 07512.

[12] Maddison WP, Maddison DR (2007) Mesquite: a modular system for evolutionaryanalysis. Mesquite v. 2.72. Available: http://mesquiteproject.org. Accessed 13 January 2013.

[13] Drummond AJ, Rambaut A (2007) BEAST: Bayesian evolutionary analysis by sampling trees. BMC Evol Biol 7: 214 17996036 10.1186/1471-2148-7-214 PMC2247476

[14] Rambaut A (2009) FigTree v1.3.1. Available: http://tree.bio.ed.ac.uk/software/. Accessed 13 January 2013.

[15] Ortiz P, Uribe C (2007) Galería de Orquídeas de Colombia (CD edition). Asociación Bogotana de Orquideología, Bogatá.

[16] Kress JW, Wurdack KNJ, Zimmer EA, Weigt LA, Janzen DH (2005) Use of DNA barcodes to identify flowering plants. Proc Nat Ac Sc USA 102 (23): 8369–8374. 10.1073/pnas.0503123102 PMC1142120 15928076

[17] Karremans AP (2010) Phylogenetics of *Stelis* (Orchidaceae: Pleurothallidinae) and closely related genera, based on molecular data, morphological characteristics and geographical distribution in the Central American and Andean Cordilleras. M.Sc. Thesis, Wageningen University. Available: http://edepot.wur.nl/146921. Accessed 22 October 2014.

[18] Karremans AP, Bakker FT, Pupulin F, Solano-Gómez R, Smulders MJM (2013) Phylogenetics of *Stelis* and closely related genera (Orchidaceae: Pleurothallidinae). Plant Syst Evol 29(1): 69–86.

[19] Bogarín D (2007) A new *Lycaste* (Orchidaceae: Maxillarieae) from Costa Rica. Lankesteriana 7(3): 543–549.

[20] Lahaye R, van der Bank M, Bogarín D, Warner J, Pupulin F, et al (2008) DNA barcoding the floras of biodiversity hotspots. Proc Nat Ac Sc USA 105: 2923–2928. 10.1073/pnas.0709936105 PMC2268561 18258745

[21] Chase MW, Williams NH, Donisete de Faria A, Neubig KM, Amaral MCE, et al (2009) Floral convergence in Oncidiinae (Cymbidieae; Orchidaceae): an expanded concept of *Gomesa* and a new genus *Nohawilliamsia* . Ann Bot 104: 387–402. 10.1093/aob/mcp067 19346522 PMC2720657

[22] Leopardi C, Carnevali G, Romero-González GA (2012) *Amoana* (Orchidaceae, Laeliinae), a new genus and a new species from Mexico. Phytotaxa 65: 23–35.

[23] Meyer CP, Paulay G (2005) DNA Barcoding: Error Rates Based on Comprehensive Sampling. PLoS Biology 3(12): e422 16336051 10.1371/journal.pbio.0030422 PMC1287506

[24] Ramos-Castro SE, Castañeda-Zárate M, Solano-Gómez R, Salazar GA (2012) *Stelis zootrophionoides* (Orchidaceae: Pleurothallidinae), a new species from Mexico. PLoS One 7(11): e48822 10.1371/journal.pone.0048822 23144987 PMC3492219

[25] Meyer GE, Basquero L, Cameron KM (2012) A new Ecuadorian species of *Dracula*: Pleurothallidinae (Orchidaceae). OrchideenJournal 19: 107–113.

